# Springhalt

**Published:** 1892-09

**Authors:** Williamson Bryden


### COMMUNICATIONS.

### SPRINGHALT.

EDITORS OF JOURNAL OF COMP. MED. AND VET. ARCH.

*Gentlemen :*—My remarks at the June meeting of the Massachusetts Veterinary Association, as reported in the August number of your JOURNAL, are somewhat misleading.

As you are aware, a portion of the spinal cord of the old horse treated by me a year ago for springhalt, was sent to "Clark University" to be examined by Prof. Donaldson; Mr. Bolton, his assistant, reported on the case at our June meeting in Boston, and demonstrated lesions found by them in their examination of the spinal cord microscopically, and which they believed to be the *cause* of the disease; this was a great disappointment to me, for while I expected that the minute examination of the cord would reveal changes in it of a sympathetic nature, I was not prepared to be told that these changes were probably the primary lesions and the *cause* of springhalt. In the discussion that followed I questioned the conclusions at which the experts had arrived, and adhered to my own original statement of the case, founded on my practical experiences in its treatment, which I had always accomplished with a fair degree of success.

I tried to point out that its relationship to *chorea*, in man or dog, must be very remote if any existed, and the fact that the spine was not treated locally, made it reasonably evident that the hoof, the only part that was treated, was the cause of the derangement.

Mr. Bolton said that Professor Donaldson thought *rest* had made the improvement which followed my treatment; as most cases of springhalt, work every day while being treated, *rest* has little influence in the matter, indeed, in many cases it is believed to aggravate the trouble especially when confined, as was the case with the little old horse.

An eminent Pathologist informs me that *spots* on the cord are found in cases of Elephuntiasis and several other diseases in man, it is therefore probable that these spots are not peculiar to springhalt only, and consequently are not the *cause* of this peculiar disease.

I regret that Mr. Bolton's paper has not been sent you, for it was a very interesting one.

With reference to our secretary's report of what I said farther, permit me to say, that I certainly do believe in "Hoof Culture," and feel rather proud of having suggested the term, for there is, in my opinion, no subject of greater inportance in the domain of equine athletics and locomotion.

I am satisfied that the horse's hoof is liable to adverse changes *that predispose to* and cause diseases of the limb above, that are peculiar to the soliped species, and that ordinary springhalt is one of this class I do not wish to be understood that accidental injury to a muscle or tendon may not, as a coincidence, cause equivocal action, very much like springhalt. I do not wish to be understood that a kick or blow at the ordinary seat of projecting bone spavin, or even a strain may not cause an exostosis, but I do contend that ordinary bone spavin as met with in the horse has a history and ætiology peculiar to this species of animal.

I object to "firing and blistering" and believe that, like "bleeding" it is doomed to a somewhat similar fate, because, if I am right, firing and blistering a spavin besides being barbarous, is treating a *result* not a *cause*, and more than that, it is treating only one of many adverse changes in the limb ; the result of the change in the hoof which causes the animal to place his weight irregularly on the extremity, is, it produces *puffs* above the ankle, *atrophies and shortening of tendons* and especially of the muscles of the hip, etc.

I treat the hoof because I believe that it dominates and causes these changes whether secondary or concurrent. It seems to me more logical and philosophic to treat the *cause*.

It might be inferred from Dr. Peter's report of my remarks that I never treat secondary or sympathetic changes of the limb ; this is a mistake, I use ordinary remedies when necessary, but always in connection with the treatment of the hoof which I consider the prime factor in the ætiology of this class of derelict conditions of the horse's limbs ; and I may add with a fair degree of success.

It did not occur to me that any one in the profession would infer that I entirely ignored these lesions of the limbs above. Yet it illustrates how some fail to grasp comprehensively the intent of those departing from orthodox lines. I have often been annoyed and humiliated at my failures to make myself understood.

Yours respectfully,

WILLIAM BRYDEN.

